# *Artemisia vulgaris* anthelmintic activities to ova and adult stages of *Fasciola gigantica in vitro*

**DOI:** 10.14202/vetworld.2023.1141-1153

**Published:** 2023-05-30

**Authors:** Andini Nurlaelasari, A’isyah Retno Wulandari, Tamara Muñoz Caro, Herjuno Ari Nugroho, Sukaryo Sukaryo, Muhammad Cahyadi, Wahyu Kurniawan, Penny Humaidah Hamid

**Affiliations:** 1 Department of Animal Science, Faculty of Agriculture, Sebelas Maret University, Indonesia; 2 Escuela de Medicina Veterinaria, Facultad de Medicina Veterinaria y Recursos Naturales, Universidad Santo Tomás, Chile; 3 National Research and Innovation Agency, Indonesia; 4 Agency of Livestock and Fishery Services, Boyolali District, Central Java, Indonesia

**Keywords:** anthelmintic, *Artemisia vulgaris*, *Fasciola gigantica*, flukicidal, ovicidal

## Abstract

**Background and Aim:**

Fasciolosis due to *Fasciola gigantica* is endemic to tropical countries and *Fasciola hepatica* in temperate climates, highly detrimental to livestock and known as foodborne zoonotic diseases. The strategic control of the disease is mainly the use of chemical anthelmintic. This study aimed to evaluate the anthelmintic properties of *Artemisia vulgaris* extract on the ova and adult stages of *F. gigantica*.

**Materials and Methods:**

Samples were collected from the Ampel Abbatoir, Boyolali District, Central Java, Indonesia. The ova from 20-gallbladders of cattle which were naturally infected with *F. gigantica* and 270 living *F. gigantica* worms were used in this study. The ovicidal assay was performed by incubating the ova with *A. vulgaris* in different concentrations, that is, 5%, 2.5%, and 1.25% for 5, 9, 11, 14, and 16 days. The efficacies were evaluated by quantification of ova degeneration during developmental stages in different time points and egg-hatch assay. The flukicidal effects were observed by mortality assay in 5, 10, 20, 40, 80, 160, 320, and 640 min incubations followed by scanning electron microscopy for surface morphology and histology of the fluke’s transversal sections.

**Results:**

The concentration of 5% *A. vulgaris* showed the strongest ovicidal activities. The percentage of hatching ova on day 16 at concentrations of 5%, 2.5%, and 1.25% were 3.33%, 6.67%, and 16.67%. These ova hatch assay showed a significant reduction (p < 0.001) compared to untreated control. The flukicidal effect was significant (p < 0.001) at a concentration of 20%, with a mortality rate reaching 66.67% in the 40 min of incubation time. The surface properties of the adult worms, including the spine, tegument, acetabulum, intestine, and vitelline follicles, were disintegrated.

**Conclusion:**

The results showed that *A. vulgaris* has the potential ovicidal and flukicidal properties to *F. gigantica*. The active compounds remained necessary to be elucidated further and its modes of action would be interesting to be predicted by molecular docking modeling.

## Introduction

Fasciolosis is caused by liver parasites of the genus *Fasciola* (Digenea: Fasciolidae) [1]. It is commonly found in tropical regions of the world, such as Asia, Africa, and the Middle East [2, 3]. Worms size ranges from 33.23 ± 6.77 to 9.59 ± 2.19 mm [4–7], and like other trematodes, the species have complex life cycles, requiring vertebrates as primary hosts for sexual reproduction. The parasite reproduces asexually within the intermediate hosts such as *Lymnaeidae* snails [2]. *Fasciola* spp. ova are shed in the environment through feces from the definitive host, and the miracidium actively looks for *Lymnaeid* snails as intermediate hosts. Infected snails release cercariae to form cysts and attach to plants as metacercariae as the infective stage of the parasite [8]. Metacercariae are characterized by hard cyst walls [9], and when ingested by ruminants, they become infected with *Fasciola* spp. [10]. The infection leads to impaired growth of productive cattle, reduced milk and meat production. Direct economic losses caused by rejection of infected livers in the abattoirs due to liver fibrosis and cirrhosis [11], and animal care costs for anthelmintic as curative or prophylaxis. The estimated economic loss due to fasciolosis with condemnation of the liver is varying, as exemplary in livestock slaughtered in Iran reached 15,831,959 USD [12].

Fasciolosis from the infections of *F. gigantica* and *F. hepatica* occurs in mammals, such as cattle, buffalo, sheep, and goats [13] and humans [14]. About 300 million cattle and 250 million sheep have been infected with fasciolosis [15]. The lowest and highest prevalence is in goats and cattle at 0.0%–47.0% and 0.71%–69.2% in 13 Asian countries [13]. Fasciolosis is endemic in countries and infects 17 million people from 61 countries, while 180 million people are at risk of infection [16, 17]. Special attention should be given to this disease because, in recent years, there has been an increase in the number of cases in humans [18]. Furthermore, several countries reported cases of fasciolosis, such as Australia [19], Brazil [20], Colombia [21], Egypt [22], Ethiopia [23], Germany [24], Indonesia [25], Iran [12], Mexico [26], Nigeria [27], Papua New Guinea [28], Peru [29], Poland [30], Saudi Arabia [31], Ukraine [32], and Zimbabwe [33]. The disease is estimated to cause 90,041 disability adjusted life years [34].

Prevention and treatment strategies are very important to avoid losses to livestock farmers [35]. The control for *F. gigantica* is commonly conducted using commercial drugs such as albendazole [36], triclabendazole [37], and nitroxynil [38]. Albendazole targets adult liver flukes [39], and triclabendazole kills both mature and immature worms [40]. The problem of drug resistance needs concern since many reports exist [38]. Reports of drug resistance have been observed in Western countries such as Australia [41], Peru [42], Northern Ireland, Wales, Scotland, New Zealand, and Spain [43]. The use of natural ingredients as an alternate anthelmintic is considered environmentally friendly and based on biological resources. Indonesia is rich in biodiversity, including herbal plants used since ancient times [44]. Chinese New Leaf Plants, *Artemisia vulgaris*, can be found in Indonesia easily [45]. *Artemisia vulgaris* is abundant in areas with a height of 3,000 m above sea level. This plant is a half-timber herbaceous plant with many branches, grooves, hair, and flowers in the open field [46]. Its essential oil has antibacterial, antifungal, and anthelmintic properties [47]. The phytochemical screening with 96% ethanol as solvent reportedly contained flavonoids, tannins, saponins, and steroids/terpenoids [48]. It is known that flavonoids have been evident to cause protein denaturation in worm tissue, which initiates worm death [49]. Saponins stimulate neuromuscular activities through the parasympathetic nerves, causing convulsions and mortalities [50]. Interestingly, *A. vulgaris* has been shown to have enormous potential as a medicinal plant to overcome helminthiasis, that is, *Haemonchus contortus* [47]. However, the potential use of *A. vulgaris* as an anthelmintic to trematodiasis has never been reported.

This study aimed to evaluate the anthelmintic properties of *A. vulgaris* extract to *F. gigantica*, the most prevalent trematode in livestock of tropical countries, on the ova and adult stages *in vitro*.

## Materials and Methods

### Ethical approval

All experiments for collecting adult *F. gigantica* from naturally infected cattle were approved by the Ethics Committee of Ahmad Dahlan University, Indonesia (No. 022206036).

### Study period and location

The study was conducted from July to November 2022. The fluke samples were collected in Ampel Abbatoir, Boyolali District, Central Java, Indonesia. The extraction of the herb was performed in LPPT, Gadjah Mada University, Indonesia. The experiments were performed in UPT Integrated Laboratory, Sebelas Maret University.

### Ova and adult stadia collections

Ova and adult worms of *F. gigantica* were collected from the Ampel Abbatoir, Boyolali District, Central Java, Indonesia. The parasites were obtained from cattle at abattoirs after a veterinarian diagnosis of macroscopically observed infection in the postmortem inspection. The gallbladders were taken into the laboratory on ice, 4°C. The samples were further processed, such as dissection and filtration to obtain ova.

Briefly, the gallbladder contents were collected in a 1000 mL beaker glass and mixed with tap water. The homogenous solutions were transferred to a 15 mL conical centrifugation tube and centrifuged at 2300× *g*. Centrifugation was carried out several times by washing steps with tap water, aquadest, and aquadest-Percoll for 10 min each. *Fasciola gigantica* ova were settled to the bottom, and the supernatant was discarded. Sedimentations were repeated until the suspension was completely clear [51]. The last rinse with aquadest-Percoll was performed using a sterile conical tube. After the isolation process was completed, the ova were stored in the refrigerator until use.

### Species identification

The collected *F. gigantica* worms were identified based on morphology by a light microscope (Olympus, Japan) at 40× magnification. Ova samples were taken by obtaining 50 μL of the suspension, transferred on an object glass, and observed. The observations were categorized into normal, degenerated, and developed [52]. The normal ova category starts morulation on the 5 days, and the larvae develop on 9^th^ day onward of incubation. The category of a degenerated egg is determined by damage to the ova wall, damage to the cell nucleus, and the operculum opening before maturation [52].

Biomolecular identification was also carried out to ensure that the ova and worms used were homogenous *F. gigantica*. The ova and worms were isolated, forming a pool containing at least 100 ova in a 1.5 mL microtube. Worm tissue pooling was also carried out on 8–10 tissue sections of individual adult worms in a 1.5 mL microtube. Following the kit protocol, the collected ova and worm DNA were isolated using the DNeasy extraction kit (Qiagen, Germany). Isolated DNA was transferred into a 1.5 mL microtube and stored at −20°C. Furthermore, the Duplex polymerase chain reaction (PCR) reaction [18] was carried out with a T100 Thermal Cycler Machine (BioRad, Singapore), and the total reaction volume in the microtube was 25 μL. The reaction composition was 12.5 μL My Taq Mix (Bioline, USA), nuclease-free water (1^st^ Base, Singapore), 2 forward primers FHF (5’-GTTTTTTAGTTTGGGGTTTG-3’), FGF (5’-TGTTATGATCATTGTTTGTAG-3’), a reverse primer FHGR (5’-ATAAGAACCGACCTGGCTCAC-3’) of 1 μL each 10 μm, and 1 μL of DNA for each sample. The PCR reaction began with pre-denaturation, denaturation, annealing, extension, and final extension at 95°C, 95°C, 52°C, 72°C, and 72°C for 180, 30, 30, 120, and 120 s, respectively. The PCR reaction was repeated for 35 cycles, and the results were electrophoresed using a submarine electrophoresis system on 1.2% agarose gel with ethidium bromide dye for 40 min at 50 volts. Thereafter, PCR visualization was performed using the Glite UV Gel Documentation System (Pacific Image, Taiwan), with the GeneRuler 1 kb DNA ladder (Thermo Scientific, USA) as standard.

### Artemisia vulgaris preparation

*Artemisia vulgaris* was collected from Temanggung, Central Java, Indonesia, with coordinates 7° 20′ 02.8″ S 110° 01′ 54.8″ E. The leaf was identified morphologically by letter no. 073/S.Tb/IV/2022 at the Faculty of Biology, Laboratory of Plant Systematics, Gadjah Mada University, Indonesia. The leaves were cleaned and dried in direct sunlight for 7 days, and dried leaves were ground into powder in a grinding machine. The powder was extracted using the maceration method at Integrated Research and Testing Laboratory, Gadjah Mada University in Indonesia. The maceration process started by adding 96% ethanol to the powder of the *A. vulgaris* plant. The mixture was stirred using ultraturaq for 30 min and soaked for 48 h. Furthermore, filtering and adding ethanol were carried out, then the filtering was repeated. The filtrate was evaporated with a vacuum rotary evaporator using a water bath at 50°C. The thickened extract was poured on a porcelain recipient, then heated in a water bath at 70°C and stirred occasionally.

### Ovicidal activities

In this experiment, 1 mL of solution contained at minimum 100 ova was incubated in 1 mL total volume of *A. vulgaris* extract with different concentrations, that is, 5%, 2.5%, and 1.25%. Fluconix-340 (Interchemie, Holland) and aquadest were used as positive and negative controls, all of the treatments were performed in triplicates. Observations of ova development were carried out on days 5, 9, 11, 14, and 16. Miracidia were stimulated to hatch on day 14^th^ by intensive light with 100-watt lamp [53].

### Adulticidal efficacies

Worms were identified as “living” based on their muscular activity and response to stimuli. Alive worms still move their heads or parts of their bodies when mechanical stimulation is given [54]. Five adult worms were soaked in each concentration of *A. vulgaris*. The incubation solution was set up to 10 mL volume, each concentration. The concentrations *A. vulgaris* and Fluconix-340 used were 40%, 20%, 10%, 5%, 2.5%, and 1.25%. The negative control was physiological sodium chloride (NaCl) 0.9%, and each treatment was repeated 3 times. Observations of worm mortality were performed at 5, 10, 20, 40, 80, 160, 320, and 640 min. Mortality was determined when the worms did not respond to mechanical stimuli [54] and recorded.

### Processing of *F. gigantica* tissue section

Histology of worms was carried out at the Anatomical Pathology Laboratory at the Faculty of Medicine, Gadjah Mada University, Indonesia. The samples of *F. gigantica* at the highest concentration and the longest observation time were analyzed for internal organ characterization by transversal tissue sections. Subsequently, the worms were transversally incised and fixed by using 10% formalin. Specimens were dehydrated with alcohol gradually and then cleared by xylene. The samples through the embedding stage were printed with paraffin, and the finished preparations were stained with hematoxylin-eosin. In the last stage, the specimens were fixed [55, 56].

### Scanning electron microscopy (SEM)

Samples of *F. gigantica* were immersed in ethanol and transferred into 15 mL tubes, then sent to the Integrated Laboratory (iLab), National Research and Innovation Agency, Cibinong, Indonesia. The effect of *A. vulgaris* extract was observed using SEM with 40,000× magnification. In the first step, the samples were washed by immersing them in cacodylate buffer for 2 h, then followed by the agitation process in an ultrasonic cleaner for 5 min. The samples were placed in 2.5% glutaraldehyde for hours. Fixation with 2% tannic acid for 6 h, followed by washing using cacodylate buffer for 5 min with four repetitions. The samples were then immersed in 50% alcohol for 5 min 4 times, 70% for 20 min, 85% for 20 min, 95% for 20 min, and finally in absolute alcohol for 10 min at room temperature (21-25°C). The samples were then dehydrated in butanol for 10 min 2 times, frozen, and then freeze-dried. The specimens were mounted on specimen stub as needed and coated with gold using ion coater, while observations were made under SEM (JEOL JSM-6510LA, Belgium).

### Ultraviolet–visible (UV-VIS) spectrophotometric assay of *A. vulgaris* extract

Samples of *A. vulgaris* extract were sent for UV-VIS spectrophotometric (Shimadzu, Japan) assay to LPPT, Gadjah Mada University, Indonesia. The compounds were tested for flavonoids, alkaloids, tannins, and saponins. The total flavonoid was measured as equivalent to quercetin. About 10.0 mg quercetin was accurately weighed, then 0.3 mL of 5% sodium nitrite and 0.6 mL of 10% aluminum nitrate were incubated for 5 min each. Furthermore, 1 M of 2 mL sodium hydroxide was added, and the volume was adjusted to 10 mL. The solution was diluted again according to the standard curve and read the absorbance at λ 510 nm. The analysis was performed by taking 50 mg of the sample and 0.3 mL of 5% sodium nitrite for 5 min. About 0.6 mL of 10% aluminum nitrate was added and allowed to stand for 5 min. Afterward, 2 mL of 1 M sodium hydroxide was added to a volume of 10 mL in a 10-time dilution, and the absorbance was read in the quercetin standard curve development procedure with 510 nm wavelength.

Total alkaloid was determined as equivalent to quinine as a standard curve. Preparation of the standard curve begins by weighing 10 mg of quinine standard, added with 5 mL of 2N HCl, then shaking and filtering. The sample for 100 mg was added with 5 mL of 2N HCl and homogeneously mixed. Meanwhile, the solution was washed with 10 mL of chloroform three times in a separatory funnel to discard the phase. The solution was neutralized by adding 0.1 N NaOH, 5 mL BCG, and 5 mL phosphate buffer. It was extracted with 5 mL of chloroform and stirred with a magnetic stirrer for 15 min. Extraction with chloroform was repeated twice, and the phase was collected, evaporated with nitrogen gas, and added with a total volume of 10 mL, while absorption was read at 470 nm wavelength.

Total tannin was determined to be equivalent to tannic acid by extracting 50 mg of the sample with 10 mL of diethyl ether for 20 h and filtering. The remaining diethyl ether was evaporated and added with distilled water to the sample to a volume of 10 mL. Furthermore, 1 mL of sample solution was taken, and 0.1 mL of folin ciocalteu reagent was added, followed by vortex for 5 min. The sample was added with 2 mL of 20% sodium carbonate and vortexed for 5 min. About 10 mL aquadest was diluted 10 times and read at the absorbance of λ 760 nm after incubating for 30 min at 21-25°C. The standard curve preparation procedure used 0.0100 g of tannic acid, 10 mL of folin ciocalteu reagent, and vortexing for 5 min. Meanwhile, 20% sodium carbonate was added to a volume of 100 mL, then diluted according to the standard curve concentration. Absorbance was read at λ 760 nm after incubation for 30 min at 21-25°C.

Determination of total saponins started with weighing ± 50 mg of sample, then added with 2 mL of 25% H_2_SO_4_. The solution was autoclaved for 120 min at 110°C and extracted with ether. The filtrate was dried and added 1 mL of water, then vortexed for 5 min before adding 2 mL of 50% sulfuric acid. The solution was heated in a water bath at 60°C for 10 min. In addition, it was put into a measuring flask, and water was added to a volume of 10 mL, then diluted 5 times. Absorption was read at 435 nm, and the saponin standard curve was prepared by weighing 10 mg of Quilaja Bark. About 5 mL of water was poured, and the extract was vortexed for 5 min; then 50 μL anisaldehyde was added, homogenized, and allowed to stand for 10 min. About 2 mL of sulfuric acid 50% was heated in a water bath at 60°C for 10 min. The solution was then put into a volumetric flask, and water was added to a volume of 10 mL. The standard was diluted from 200, 100, 50, 25, 12.5, and 6.25 μL, and the absorbance was read at 435 nm.

### Statistical analysis

Statistical analyses and graphical visualizations were performed with GraphPad Prism 8.0 (GraphPad Inc. USA).

## Results

### Identification of ova and adult stages of *F. gigantica*

**Figure-1:**
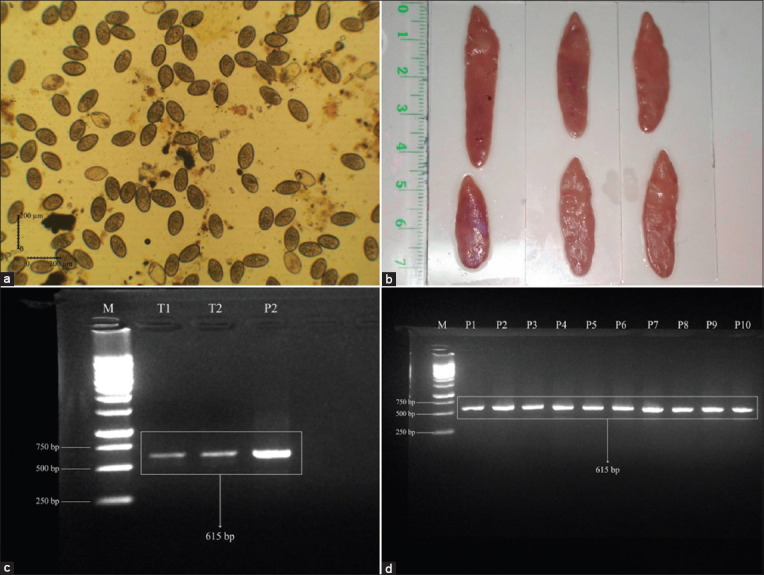
Screening of *F. gigantica* population on adult and ova stages by duplex polymerase chain reaction (PCR). (a) Ova from freshly dissected gallbladders. (b) Adult *F. gigantica* from the liver parenchyma. (c) Pooled ova *F. gigantica* by duplex PCR. (d) Pooled adult *F. gigantica* by duplex PCR.

Ova were successfully collected from 20-gallbladders of naturally infected cattle (Figure-1a), and adult worms of *F. gigantica* were obtained from liver parenchyma (Figure-1b). The intact ova were indicated with an ovoidal shape with a closed operculum at one pole. Likewise, ova showed a thin shell and a blastomere could be found filling the cavity [57].

The result of duplex PCR from the ova and worms pool showed a homogenous *F. gigantica* as showed by a single band of 615 bp and interpreted that the entire population used was *F. gigantica* (Figures-1c and d).

### Ovicidal efficacy of *A. vulgaris* to *F. gigantica* ova

**Figure-2:**
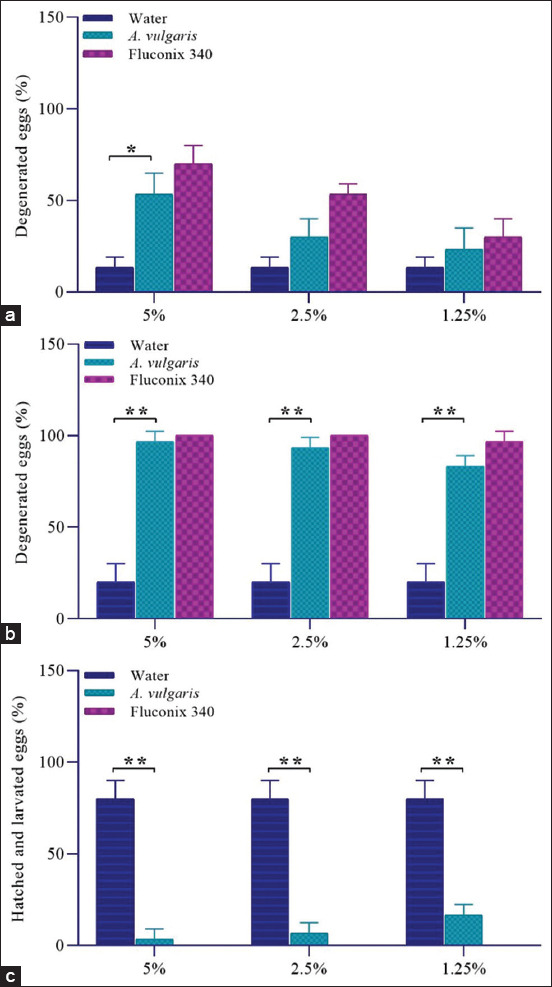
Ovicidal activities of *A. vulgaris* at different concentrations and incubation time compared to negative control (aquadest) and positive control (Fluconix-340). (a) Degenerated ova after incubation on day 9. (b) Degenerated ova after incubation on day 16. (c) Total number (%) of hatching ova on day 16^th^. *Asterisks* indicate statistical significance at the p < 0.05 level (*), statistical significance at the p < 0.001 (**).

**Figure-3:**
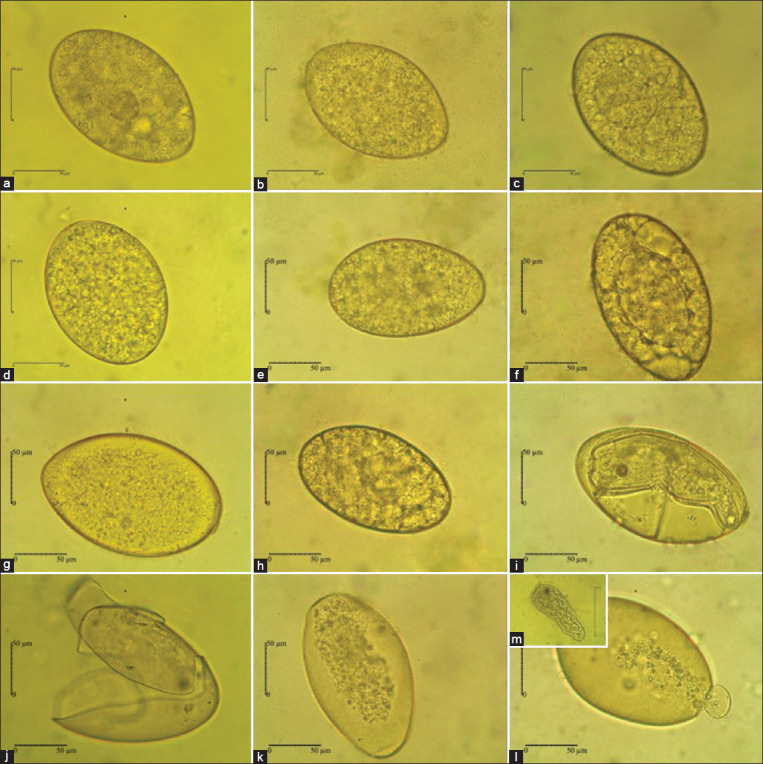
Ova development of *Fasciola gigantica* showed different morphology during treatment in (a-c) 5 days; (d-f) 9 days; (g-i) 14 days; and (j-m) 16 days. Ova were incubated in Fluconix-340 (a, d, g, j), *Artemisia vulgaris* (b, e, h, k), and aquadest (c, f, i, l, m).

Ova incubation and maturation were successfully performed until miracidia hatch. The ova were incubated at normal laboratory room temperature (21°C–25°C) without light for 14 days [52]. The percentage of degenerated ova on day 9 for the negative control was 13.33%, while for the positive control at a concentration of 1.25% was 30%, 2.5% was 53.33%, and 5% was 70% (Figure-2a). The degenerated ova treated with *A. vulgaris* extract at a concentration of 1.25% was 23.33%, 2.5% was 30%, and 5% was 53.33%. Data analysis showed that at 5% concentration, *A. vulgaris* significantly had the ovicidal effect (t = 5.36, p < 0.05), but not statistically significant at concentrations of 2.5% (t = 2.5, p = 0.06) and 1.25% (t = 1.34, p = 0.25). The observations on the 16 days of incubation showed an increase in the percentage of degenerated ova when compared to the 9^th^ day (Figure-2b). The percentage of degenerated for negative control was 20%, while for positive control at a concentration of 1.25% was 96.67%, 2.5% was 100%, and 5% was 100%. Incubation with *A. vulgaris* showed degenerated ova at concentrations of 1.25%, 2.5%, and 5% were 83.33%, 93.33%, and 93.33%, respectively. Data analysis showed that at all concentrations, *A. vulgaris* had very significant results, with concentrations of 1.25% (t = 9.5, p < 0.001), 2.5% (t = 11, p < 0.001), and 5% (t = 11.5, p < 0.001). Embryo development in the Fluconix-340 as drug-positive control was failed, as indicated by undeveloped ova and degenerated ova (Figures-2c and 3a, d, g, j). The degenerated ova were indicated by empty ova (Figure-3g), broken ova wall (Figure-3j), and dead ova with lysed morula (Figure-3k). The treatment of *A. vulgaris* was able to degenerate and inhibit the development of embryos, as indicated by reduced numbers of hatched miracidia (Figures-2c and 3b, e, h, k). Embryo development in negative control was successful, as indicated by fully developed miracidium and hatched miracidia on the incubation medium on day 14 onward (Figures-3c, f, i, l).

### Flukicidal effect of *A. vulgaris* on adult *F. gigantica*

**Figure-4:**
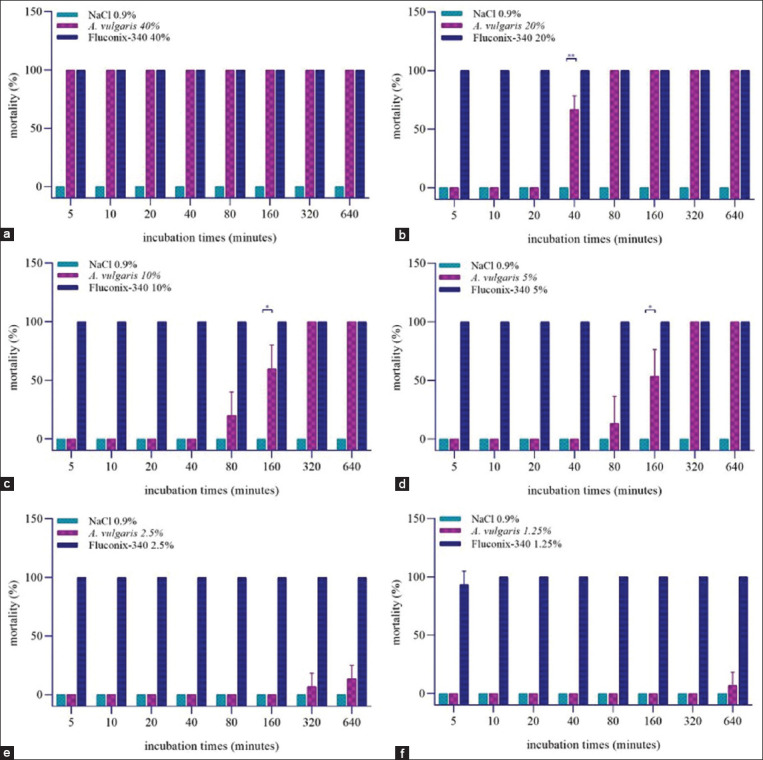
Flukicidal efficacy of *Artemisia vulgaris* against adult worms *Fasciola gigantica*. Treatment with immersion sodium chloride 0.9%, *A. vulgaris*, and Fluconix-340 at concentrations of (a) 40%, (b) 20%, (c) 10%, (d) 5%, (e) 2.5%, and (f) 1.25%. *Asterisks* indicate statistical significance at the p < 0.05 level (*), statistical significance at the p < 0.001 level (**).

**Figure-5:**
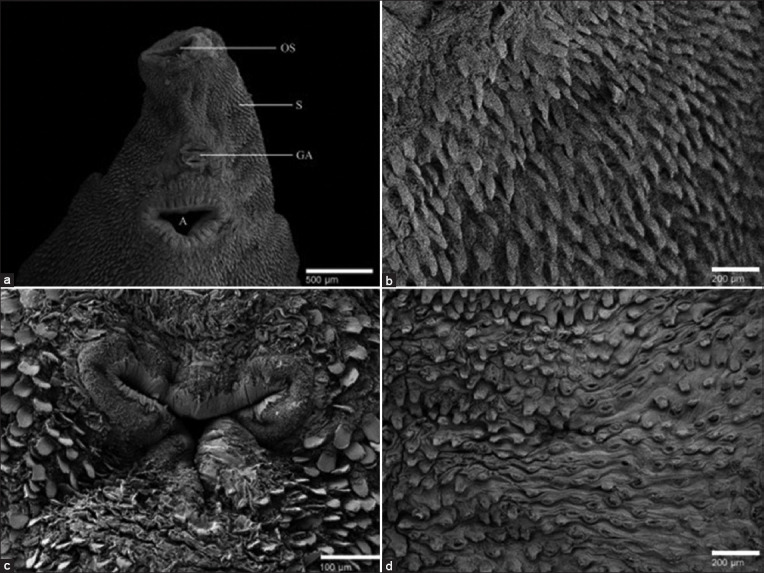
Scanning electron microscopy of *Fasciola gigantica*, (a) Anteroventral region of negative control: OS - Oral succer, A - Acetabulum, GA - Genital atrium, and S - Spina. (b) Ventral spina of negative control. (c) Ventral sucker treated with 40% *Artemisia vulgaris*. (d) Ventral spina of treated sample with 40% *A. vulgaris*.

A total of 270 adult worms was used in the experiment. The negative control with Sodium chloride 0.9% did not show any dead worms until the 640 min incubation (Figure-4). In the positive control treated with Fluconix-340, worms mortality reached 100% at first 5 min for incubation in the concentration of 40%, 20%, 10%, 5%, and 2.5%. At the concentration of 40% *A. vulgaris*, worm mortalities reached 100% starting from 5^th^ min (Figure-4a). In 20% *A. vulgaris*, worms mortality reached 66.67% at 40 min and 100% at 80 min. The flukicidal effect of *A. vulgaris* 20% was very significant (t = 10, p < 0.001) compared to negative control after 40 min of incubation (Figure-4b). Worm mortality decreased at a concentration of 10% *A. vulgaris*, when compared to a concentration of 20% *A. vulgaris*. In 10% *A. vulgaris*, worms mortality reached 33.33% at 80 min, 60% at 160 min, and 100% at 320 min. The flukicidal effect of *A. vulgaris* 10% was statistically not significant (t = 1.73, p = 0.15) compared to negative control at 80 min, but significant (t = 5.19, p < 0.05) at the 160 min of incubation (Figure-4c). Worm mortality in 5% *A. vulgaris* began to occur in the 80^th^ min reaching 13.33%, 53.33% at 160^th^ min, and 100% at 320^th^ min. Data analysis showed a significant result at 160^th^ min (t = 4, p < 0.05), but not significant at 80^th^ min (t = 1, p = 0.37) (Figure-5d). In 2.5% *A. vulgaris*, worms mortality started in the 320^th^ min reaching 6.67%, and 13.33% at 640^th^ min. The flukicidal effect of 2.5% *A. vulgaris* was not statistically significant (t = 1, p = 0.37) and (t = 2, p = 0.11) both at the 320 and 640 min (Figure-5e). At the last concentration, Fluconix-positive control 1.25% only reached 99.33% mortality compared to the previous concentration which worm mortalities always reached 100% starting from 5^th^ min. Meanwhile, 1.25% of *A. vulgaris* began to cause death at 640 min by 6.67%. Data analysis showed that the results were not statistically significant (t = 1, p = 0.37) compared to negative control (Figure-5f).

### Spectrophotometric test of *A. vulgaris* ethanol extract

The test results of *A. vulgaris* extract using UV-VIS spectrophotometric found that the content of tannins, flavonoids, saponins, and alkaloids were 15.24 % b/b, 3.41 % b/b, 2.59 % b/b, and 0.17 % b/b, respectively.

### Surface and internal organs change of *F. gigantica*

Scanning electron microscopy of *F. gigantica* is presented in Figure-5. The negative control showed the ventral region of the worm had a normal oral sucker, acetabulum, genital atrium, and tegument (Figure-5a). Normal tegument infoldings indicated a well-distributed intact and sharp spine, no swelling, and without furrowing in the surface body of the *F. gigantica* (Figure-5b). In samples treated with 40% *A. vulgaris*, it was characterized by the severe invagination of the acetabulum (Figure-5c), and a large number of spine appeared to be disrupted or eroded from the tegumental surface, leaving holes at the attachment location (Figure-5d).

**Figure-6:**
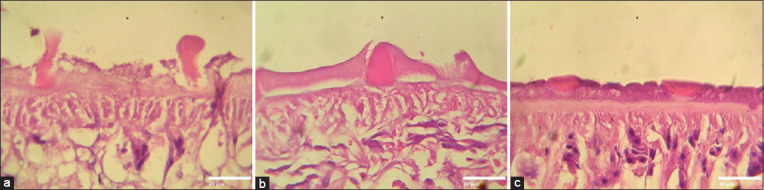
Hematoxylin-eosin staining of worm section. (a) Tegument of *Artemisia vulgaris* treated sample. (b) Tegument of Fluconix-treated sample. (c) Tegument of untreated *Fasciola gigantica*. Scale bars are 40 μm.

The effect of *A. vulgaris* on the tegument was demonstrated by cross-sectional worms stained with hematoxylin-eosin (Figures-6a-c). In the negative control, tegument syncytium was intact and well attached to the basal lamina (Figure-6c). Meanwhile, tegument syncytium of *A. vulgaris* treated sample had it distal cytoplasm destruction, which causes the detachment of spina (Figure-6a). In the Fluconix-treated sample, some of the tegument syncytium detached from the basal lamina due to vacuolation (Figure-6b).

**Figure-7:**
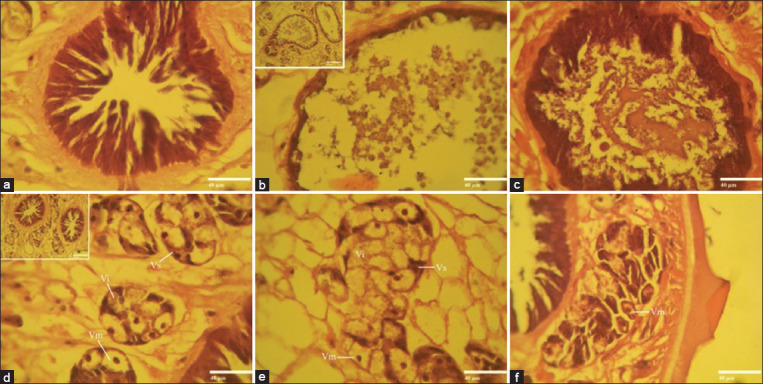
Hematoxylin-eosin staining for cross section of *F. gigantica* intestine with: (a) Sodium chloride-negative control, (b) *A. vulgaris* treated samples, and (c) Fluconix-positive control. Profile of vitelline follicles: (d) Sodium chloride-negative control, (e) *A. vulgaris* treated samples, and (f) Fluconix-positive control. Vs - Stem cells, Vi - Early or late intermediate cells, and Vm - Mature vitelline cells. Scale bars are 40 μm.

The intestinal histology was intact for untreated *F. gigantica* (Figure-7a). While, the intestinal appearance of the treated worms with 40% *A. vulgaris* showed intestinal epithelium rupture and loss of almost all villi (Figure-7b). In Fluconix-treated sample also showed intestinal epithelium rupture but the villi were still attached (Figure-7c). Vitelline follicles in control worms were normal in shape, with a well-represented vitelline cell at all stages, that is: Stem, early or late intermediate, and mature vitelline cells (Figure-7d). Vitelline follicles treated with 40% *A. vulgaris* indicated fewer mature cells, and the wall outline was less clear than controls (Figure-7e). Meanwhile, the vitelline follicles in Fluconix-340 experienced cell shrinkage, and mature cells remained intact (Figure-7f).

## Discussion

A total of 17 million people from 61 countries are infected with *Fasciola* spp., while another 180 million are at risk of infection [2, 17]. The estimated economic loss due to fasciolosis with condemnation of the liver is varied, as exemplary in livestock slaughtered in Iran reached 15,831,959 USD [12], with cases occurring on several continents, including temperate and tropical climates. One of these losses occurred for costs related to treatment with anthelmintics. In contrast, treatment using anthelmintics has been widely reported to have resistance [38, 41–43]. In this regard, using herbs as anthelmintics may provide alternative, non-toxic, and be expected to have minimum side effects, as has been reported [58]. *Artemisia vulgaris* contains compounds with potential activities against diseases, such as flavonoids, tannins, saponins, and steroids/terpenoids [48]. These substances are reported to be effective as an insecticide [45], anti-cancer [59], anti-oxidant [60], and antibacterial [61]. Anthelmintic studies of *A. vulgaris* have been carried out on earthworms [62], but the evaluation as an anti-trematode has never been reported. In this study, *A. vulgaris* was successfully demonstrated to have both ovicidal and flukicidal activities to *F. gigantica*.

We showed here that *A. vulgaris* inhibited ova maturation and reduce the number of successfully hatching miracidia. During the developmental stage, ova treated with *A. vulgaris* showed delayed morulation compared to negative controls. Almost all ova walls remained intact until 16 days of incubation. The ovicidal effect was seemed different from ova treated with Fluconix-340, which showed disintegrated ova wall more frequently. The majority of ova treated with *A. vulgaris* did not undergo blastulation and therefore, organogenesis failed in time; it should be at room temperature (25-27˚C) [63, 64] and compared by negative control development in this study (Figure-3). Thereafter, failure during development stages consequences with a significantly lower number of hatching miracidia in all concentrations tested. Our isolate *A. vulgaris* in this study contains 15.24% b/b of tannins, 3.41% b/b of flavonoids, 2.59% b/b of saponins, and 0.17% b/b of alkaloids. Another study regarding the content of *A. vulgaris* is also known to contain tannins, flavonoids, saponins, and alkaloids [65]. These contents may contribute to the ovicidal properties shown in this report. Similarly, extract *Moringa oleifera* is reported to have chemical components of flavonoids, alkaloids, tannins, and saponins [66, 67], which caused the majority of *F. gigantica* ova to become non-embryonated and inhibited the development of blastomeres [52]. Ethanolic extract of *A. vulgaris* with equal concentration exhibits higher ovicidal activity to *F. gigantica* ova than 50 mg/mL ethanolic extract of *M. oleifera* [52]. In lower concentration, 12.5 mg/mL *A. vulgaris* still showed a high effect with 83.33% undeveloped ova. Ovicidal activities of several herbs to *Fasciola* spp. were reported in different concentrations and incubation times. *Calotropis procera* in 4 mg/mL induced 81.2% ovicide by 14 days of incubation. However, it was not mentioned the species of *Fasciola* spp. ova used for the study [68]. In our experiment, we selected species populations based on the molecular screening to ensure *F. gigantica* ova were solely used. It is unknown that the same herbs would have a similar ovicidal property when tested against different species of *Fasciola* spp., that is, *F. gigantica* and *F. hepatica*. It is noteworthy that some herbs containing phenolics, tannins, and terpenoids, that is, *Eugenia uniflora* L., *Psidium guajava*, and *Harpagopytum procumbens* possess ovicidal properties to *F. hepatica* [69].

In this study, 100% *F. gigantica* lost their motility when incubated in 40% *A. vulgaris* in around 5 min incubation time, in which we counted as a mortality rate. Worm loss motility can be caused by actin-myosin disorders, impair muscle performance, and thus paralysis [70]. The tegumental changes in our SEM observation may contribute to this motility disability. Severe tegument disruption is proposed to involve in disruption of cytoskeleton and Na^+^/K^+^ ion pump [71]. Excess Na^+^ ions enter the syncytium and are pumped into the basal lamina, resulting in hypertonicity around the cytoplasm, forcing the syncytium tegument to detach from the basal lamina. It is known that changes in worms’ tegument is included in the parameter of anthelminthic activity [72].

Tegumental erosions were clearly seen in *A. vulgaris* treated worms. It may presumably introduce more substances to be internalized to deeper tissues and lead to internal organ disruptions. The intestinal epithelium rupture may occur due to the saponin component contained in *A. vulgaris*. When saponins interact with cell membranes, it can affect the cell to be more permeable and consequences with macromolecules imbalance which lead to cell lyse [73, 74]. In addition to the presence of tannins, saponins, alkaloids, and flavonoids, which are suspected of having a role in anthelmintic properties, *A. vulgaris* contains artemisinin as previously reported [75]. Artemisinin is produced from the Artemisia genera, which has been reported as anti-malarial, anti-cancer, anti-inflammatory, anti-parasitic, and antifungal [76–79]. In worms, artemisinin has been shown to act as an anthelmintic in both *in vivo* and *in vitro* studies of *Trichinella spiralis* [80]. No report or effect of *A. vulgaris* on *F. gigantica* so far. However, the substances involved in other worm genera may be included in this study. In this study, *A. vulgaris* possesses very promising activities to *F. gigantica* both in ova and adult stadia. Various substances may involve and have synergistic effects on *F. gigantica*. Further study is necessary to analyze the depth of each substance to *F. gigantica*.

## Conclusion

The ethanolic extract of *A. vulgaris* has both the ovicidal and flukicidal properties of *F. gigantica*. *Artemisia vulgaris* significantly reduced hatching ova on day 16 at concentrations of 5%, 2.5%, and 1.25% compared to the untreated samples. The flukicidal effect was significant (p < 0.001) at a concentration of 20%, with morphologically worm disruptions leading to parasite mortality. The use of natural herbs with anthelmintic potentials is beneficial as eco-friendly substances for sustainable livestock farming. The active compounds remained necessary to be elucidated further and its modes of action would be interesting to be predicted by molecular docking modeling.

## Authors’ Contributions

PHH, MC, WK, HAN, and TMC: Conceptualization. PHH: Funding acquisition. AN, ARW, SS, TMC, HAN, MC, WK, and PHH: Investigation and wrote and edited the manuscript. All authors have read, reviewed, and approved the final manuscript.
